# Proteome profiling of embryo chick retina

**DOI:** 10.1186/1477-5956-6-3

**Published:** 2008-01-22

**Authors:** Mina Mizukami, Takashi Kanamoto, Nazariy Souchelnytskyi, Yoshiaki Kiuchi

**Affiliations:** 1 Department of Ophthalmology and Visual Science, Graduate School of Biomedical Sciences, Hiroshima University, Hiroshima, Japan; 2 Karolinska Biomics Center, Dept. Oncology Pathology Karolinska University Hospital, Stockholm, Sweden

## Abstract

**Background:**

Little is known regarding the molecular pathways that underlie the process of retinal development. The purpose of this study was to identify proteins which may be involved in development of retina. We used a proteomics-based approach to identify proteins that are up- or down-regulated during the development of the embryo chick retina.

**Results:**

Two-dimensional gel electrophoresis was performed with the retina of embryo chicken, which was obtained from embryos of day 7 (ED7) and of day 11 (ED11). The protein spots showing significant differences were selected for identification by MALDI mass spectrometry. Thirteen proteins were differentially expressed; seven proteins were up-regulated in embryo retina of chicken at ED 11 and six proteins were down-regulated. Significant proteins were also evaluated in embryo day 15 (ED15). Some of identified proteins were known to regulate cell proliferation, cell death, transport, metabolism, organization and extracellular matrix, and others also included novel proteins.

**Conclusion:**

We identified thirteen proteins which differentially expressed in embryonal retina of chicken at day 7, as compared to the retina of embryo of day 11. They were various regulatory proteins for cellular signaling.

## Background

Chick retina has been extensively employed as a model of retinal cell differentiation. In an chick embryo, mitosis of retinal progenitor cells begins on embryonic day (ED) 3. A part of them give rise to non-photoreceptor cells and others to photoreceptors during the interval between ED6 and ED8. Therefore, it is proposed that ED6 to ED8 is suitable for experiments aimed at analyzing mechanism of retinal differentiation. [1]

The first cells generated from retinal progenitor cells are retinal ganglion cells (RGCs). Several proteins have been found to affect development of retina. *Notch *and basic helix-loop helix protein (bHLH) play a role in neural determination and differentiation of RGC [2,3]. Another protein, Brn3 was found to be important for terminal differentiation of RGC precursors [4] Neurite outgrowth in RGCs can be promoted by E-cadherin [5], and RGCs axons are navigated by Ephrin and Ephrin receptors [6,7]. On the other hand, PaxL had an inhibitory effect on ganglion cell development [8]. Overexpression of brain-derived neurotrophic factor (BDNF) promoted differentiation of photoreceptor cells via TrkB in early chick retina [9]. Activin is associated with the differentiation to amacrine cells until ED8 and it was reported that overexpression of follistatin, an activin binding protein and an inhibitor, cause a decreased in the frequency of amacrine cells generation during chick retina development [10]. Despite reported findings, the mechanism of retina development is not fully understood. Thus, comprehensive studies are required to describe proteins whose change during retina development.

Here, we performed proteome profiling of chick retina to identify proteins that are differentially expressed between ED7 and ED11 in chick retina. We report thirteen proteins whose expression is changed in embryonal retina of chick.

## Results

### Two-dimensional proteome maps of embryo retina of chicken

**Figure 1:**
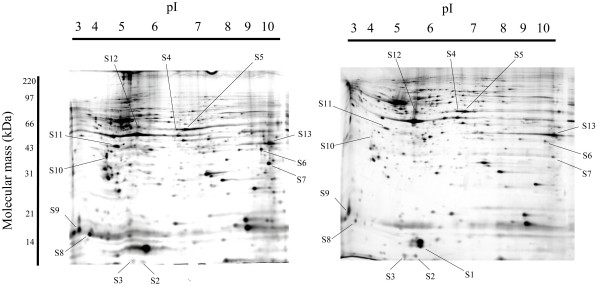
Photographs of two-dimensional electrophoresis gels with annotation of the spots of identified proteins. The left image shows a silver-stained gel of embryo chick retina at ED7 and the right image is that of embryo chick retina at ED11. The proteins spots that increased or decreased in embryo days ED7 to ED11, and that were identified by PMF are shown. Spots S1 through S13 represent the annotated spots. The pI gradient of the first dimension electrophoresis is shown on the top of the gels, and the migration of molecular mass markers for SDS-PAGE in the second dimension is shown on the side of the gel. Representative gel images are shown.

To identify the development-dependent proteins in the retina, we compared the proteome of ED7 chick retina with that of ED11. Total lysates of retina samples were resolved by two-dimensional gel electrophoresis. We detected an average one thousand protein spots on the two-dimensional gels after silver staining (Figure 1). We generated three gels for each experimental condition to ensure the reliability of the selection of spots with significant changes of expression. [See Additional file 1]

A mother gel was constructed using images of three gels of chick retina by PD-Quest software. The volume of all of the protein spots was quantitatively analyzed, and the maximum volume of a single spot was 46,685 arbitary unit (a.u.). Weak spots, whose volumes in the matched pair spots were both less than 5000 a.u., were deleted to avoid the small change of the paired spots. We removed from analysis spots which showed volume difference of less than two fold between compared conditions, i.e. ED7 vs ED11. Finally, twenty spots that had increased or decreased volume in embryo chick retina were selected as significant spots and MALDI TOF mass spectrometry was performed to identify the proteins. [see Additional file 2] Thirteen proteins were identified in selected spots. Furthermore, protein expressions of significant spots were also evaluated in embryonic day 15 (ED15).

### Clustering of identified proteins

Analyses of the identified proteins showed that embryonic retinal development is required for the potential of altering different cellular functions (Table 1). We found that the expression of seven of the thirteen proteins (54%), S1 through S5, S12, and S13, was increased in ED11 of embryo chick retina and six (46%), S6 through S11, were decreased (Table 1). The expression of S1 protein was undetectable in ED7 of embryo chick retina and was defined as zero in the PD-Quest-based analyses. Another twelve proteins, except for S1, were expressed in both ED7 and ED11 of embryo chick retina.

**Table 1:** Differentially expressed proteins identified by proteomics from retinas of ED7, ED11 and ED15 of embryo chick retina.

						Theoretical value		Experimental value		Changes(ratio)
	Protein	Est'Z	Taxonomy	Sequence coverage(%)	ncbi ID	pH	Mr (kDa)	pH	Mr (kDa)	ED7:ED7:ED15
S1	Fatty acid-binding protein, retina	2.13	Chick	61	Q05423	5.6	15.02	5.5	15	(-):(+):(+)
S2	Wnt-9	0.98	P. hagfish	37	P28124	11.3	15.06	5.75	10	1:14:4.6
S3	Ubiquitin like protein, SMT3A	0.4	Human	24	P55854	5.3	11.68	7	10	1:9.6:1.2
S4	unnamed protein product	0.66	Mouse	12	BAC32207.1	5.3	80.05	6.75	70	1:2.4:2.3
S5	Alpha enolase	2.38	Chick	35	P51913	6.2	47.63	6.5	100	1:2.2:1.7
S6	Sin3B	0.76	Human	10	AAH05113	6.6	49.62	9	45	2.1:1:0.3
S7	hypothetical protein	1.03	Human	23	CAD38885.1	9.2	26.71	9	35	2.5:1:1.7
S8	unnnamed protein product	0.99	Human	44	BAB14762.1	5.5	29.35	4	20	2.4:1:1.7
S9	unnamed protein product	1.51	Human	43	BAB85061.1	5.4	17.7	4	20	4.2:1:1.6
S10	ZZ type zinc finger containing protein	0.52	C. elegans	15	NP498699.1	4.5	64.85	4.5	30	7.5:1:4.2
S11	GABA receptor-A beta-3	0.61	Chick	12	P19019	9.4	54.76	5	45	2.8:1:4.1
S12	Flice	0.72	Mouse	18	CAA04196.1	5.3	49.35	5	70	1:2.9:(-)
S13	Keratin-9	2.43	Human	36	NP000217.1	5.1	62.2	9	45	1:2.3:4.0

S1 – S13 means ID numbers of spots. Taxonomy category was used for search. Probability, Est'Z, sequence coverage, and theoretical value of pI and Mr were obtained from the Pro Found search. The calculation of experimental pI and Mr was based on the migration of the protein in a 2D gel. Changes indicate the ED7 chick retinal protein volume ratio compared with that of ED11 and ED15. (-) indicate that protein spots were not detected

Fatty acid binding protein-retina (R-FABP) (S1) and Gamma-aminobutyric-acid (GABA) receptor-A (S11) has been already identified as a signaling protein and related to the development of embryonic chick retina. These findings demonstrated that our approach not only detect previously suggested functional links of retinal development, but also confirmed the validity of our technique. The identification of novel proteins opens for the possibility of novel mechanism on development of the embryo retina.

In ED15, an expression of flice (S12) in ED15 was undetectable by PD-Quest software analysis.

## Discussion

We used the proteomic approach and detected eighteen proteins that changed their expression in retina ED7 as compared to ED11. (Table 1) Known functions of some of the identified proteins suggest that they may be important for retinal development.

R-FABP (S1) is expressed in the neuritis of ganglion cells, inner nuclear layer, inner plexiform layer, optic nerve fiber layer, and non-pigment ciliary epithelium [11] and supposed as an important component of developmental program in chick retina [12]. R-FABP includes a binding site of AP-2, a repressor of R-FABP [13]. R-FABP mRNA is highly expressed in ED5 of chick retina and that is down-regulated in ED10 and vanished in ED16 [14]. But, our study showed that protein expression of R-FABP was up-regulated in ED11. Therefore, it is speculated that R-FABP protein is degraded or not translated around ED7, and that mRNA of R-FABP may be possibly down-regulated as negative feedback mechanism.

It has been known that Wnt family have an important role in the development of chick retina. Wnt 13 is required for the induction and maintenance of extraocular mesenchyme [15]. Wnt4 and Wnt5 seem to participate directly in axon guidance and Frizzled-related protein 1 (SFRP1) has the ability to guide growth cone movement via Frizzled-2 receptor [16]. Wnt9 (S2), that is expressed in mouse embryo from 9.5 to 17.5 days [17], was identified as a protein whose expression was up-regulated in ED 11 in this study. Wnt14, homologous gene to Wnt9, was closely linked to Wnt3 [18] and Wnt3 increase proliferation of dedifferentiated Muller glia [19]. Wnt2b play a role in determining the identity of ciliary body and iris derived from the peripheral optic cup and beta-catenin inhibit retinal progenitor gene expression [20]. The degradation of beta-catenin is promoted by SMT3A (S3) that is ubiquitin like protein [21] and identified in this study. Wnt signaling pathway is suppressed by histone deacetylase 1 (HDAC1), and that is required for the switch from proliferation to differentiation in zebrafish retina [22]. The activity of HDAC1 is regulated by Sin3B (S6) [23] that was identified as the protein whose expression was decreased in ED11 in this study. These speculate that Wnt9/14, Wnt3, SMT3A, and Sin3B may cooperate and play a role in the development of retina.

GABA is one of the metabolic substrates forming the metabolic cycle of glutamate that is taken in by cells [24]. GABA-B receptors regulate chick retinal calcium waves occurring before synapse formation in the embryonic chick retina [25] and calcium entry into cells is important for the regulation of neurite outgrowth in developing chick retina from ED6 [26]. GABA-A receptor does not contribute to calcium wave production [27,28] and our result showed that GABA receptor-A beta-3 subunit (GABAR-A beta3) (S11) was down-regulated in ED11. The immunofluorescence signal detected with specific antibodies against GABAR-A beta3 in the inner plexiform layer of the developing chick retina [29], and GABAR-A beta3 was also expressed in the postnatal retina of rabbits [30,31] and ferrets. [32] GABA-C receptor binds with ZIP3, which is expressed in the retina, and ZIP3 contains a ZZ type Zinc finger domain [33]. ZZ type zinc finger containing protein (S10) regulates hypertensive to red and blue 1 (HRB1) in the photomorphogenic development [34]. Thus, GABA receptors and ZZ type zinc finger containing protein may be possibly associated with the synaptic formation of retina.

Flice (S12), also called caspase-8, was expressed in axotomized retinal ganglion cells (RGCs) [35] and flice is activated in 7-keratocholesterol-induced apoptosis in R28, retinal precursor cells [36]. Flice also control the death or survival of RGCs in a rat model of choronic glaucoma [37]. We found that flice was expressed in the developmental retina of chicken and up-regulated in ED11 and disappeared in ED15. These suspect that flice have a role in the decision of programmed death of RGCs during the retinal development.

Alpha-enolase (S5) was identified and up-regulated in ED11. It was reported that alpha-enolase was an important crystalline in the chicken lens [38], but the function of alpha-enolase for the retinal development is unkown. Among unnamed proteins, S4 (BAC32207) coded kinesin superfamily protein 20B [39]. Kinesines are microtubule-dependent motor proteins that are responsible for the distribution of various organs. [40] Kinesine family 2 (KIF2) localized in retina and retinal pigment epithelium [41] and Kinesine family 3 (KIF3) localized in photoreceptor [42]. Dynactin, a regulator of the microtubule motor proteins, is required to maintain the position of the nucleous within post mitotic photoreceptor neurons. [43] and Disabled 1 (Dab1) also determines nuclear positioning in neurons [44]. These may speculate the association of Kinesine family and related proteins have an effect on the retinal development. Other unnamed proteins, S9, S10, and S11, included no domain or structure that have been reported as any relation with the retinal development previously.

## Conclusion

We report here identification of thirteen proteins which change expression during development of embryo chick retina. Identified proteins included various cellular functions.

## Methods

### Animals

Fertilized White Leghorn chicken eggs were obtained from Hiroshima Experimental Animals Ltd. (Japan). All eggs were maintained in air at 37°C and handled in accordance with the Guide for the Care and Use of Laboratory Animals by the USA National Institutes of Health.

### Sample preparation

The eyes were directly enucleated from embryo chicken. The retinas were carefully isolated from the choroid in phosphate-buffered saline (PBS). Eight retinas from eight eyes, including four right eyes and four left, at the age of ED7 were collected in one tube. Retinas at the age of ED11 and ED15 were also collected as same, and lysed with 8 M urea by pipetting. After centrifugation, the supernatant was collected to one tube as a master lysate.

Master lysates were divided two samples. One was used for measurement of protein concentration, and resolved in urea mix [8 M urea, 4% NP-40, 2% ampholine, 2% mercaptoethanol]. Protein concentration was measured by the modified Bradford protein assay [45]. Others were solubilized in sample buffer [8 M urea, 4% CHAPS, 0.5% dithiothreitol (DTT), IPG buffer, pH 3–10] for two-dimensional electrophoresis.

### Two-dimensional electrophoresis

Two-dimensional electrophoresis and protein identification were performed as described early [46]. Samples, including 50 μg protein, were applied by the rehydration technique. Isoelectrofocusing was performed on the strips with an immobilized pH gradient (pH 3–10 non-linear gradient, 18 cm: GE Healthcare). First-dimension isoelectrophoresis was performed in IPGphor (GE Healthcare) according to manufacturer's instructions. After the isoelctrofocusing, the strips were placed in equilibration buffer-1 (50 mM Tris-HCl, pH 8.8, 6.0 M urea, 2.0% SDS, 30% glycerol, 1% DTT) and then in equilibration buffer-2 (50 mM Tris-HCl, pH 8.8, 6.0 M urea, 2.0% SDS, 30% glycerol, 4% iodoacetamide). The equilibrated strips were loaded onto SDS-containing 12% polyacrylamide gel, and SDS-polyacrylamide gel electrophoresis (PAGE) was performed.

After the electrophoresis, the gels were fixed in 7.5% acetic acid and 20% methanol, and sensitized in 25% ethanol, 0.2% sodium thiosulfate, and 3.4% sosium acetate. The gels were then stained with 0.25% silver nitrate and developed with 2.5% sodium carbonate and 0.04% formaldehyde.

### Gel analyses

Silver-stained gels were scanned by an image scanner (EPSON) and analyzed with calculation of the volumes of the spots with the PDQuest (2-D Analysis Software Version.1: BioRad) following the manufacturer's instructions. The software included the equipment to correct and standardize automatically the difference of total and whole staining of the compared gels. Three gels from each type of chicken were prepared and a master gel was generated for each type of chicken. The values of the volume of each matched spot on the master gels were compared. Spots with differences in expression were then identified by mass spectrometry.

### Protein identification

The excited protein-containing spots were de-stained with 30 mM potassium ferricyanide and 100 mM sodium thiosulfate. Then, the gel pieces were dipped in 0.1 M sodium hydrocarbonate and washed with acetnitril. After the gel pieces were dried, in-gel digestion was performed with trypsin. Then, 10% trifluoroacetic acid (TFA) and acetonitrile were used to extract the peptides, and the extract was desalted on a nano-column. After washing the column with 0.1% TFA, the matrix was eluted with acetnitril containing alpha-cyano-4-hydroxycinnamic acid directly onto the MALDI target. Spectra were generated on a Bruker Biflex 3 MALDI-TOF-MS (Bruker Daltonics). The spectra were internally calibrated using known internal tryptic peptides from trypsin and searches were made in the NCBI sequences using ProFound. No restrictions on species and pI were applied, and tolerance was set on less than 0.5 Dalton. The search results were evaluated by considering the probability, the Z-value, peptide coverage, and correspondence to experimental pI and molecular mass.

## Competing interests

The author(s) declare that they have no competing interests.

## Authors' contributions

MM carried out the proteomic studies. TK carried out the proteomic studies, data analysis, and participated in drafting the manuscript. NS carried out data analysis. YK participated in drafting the manuscript.

## Supplementary Material

Additional file 1 Actual photographs of two-dimensional electrophoresis gels. Upper three panels showed silver-stained gels from ED7, middle from ED11, and lower from ED15. Click here for file

Additional file 2 Protein volume of significant spots. The data provide the actual protein volume (arbitary unit; a.u.) and SEMs of significant protein spots, measured by PD-Quest software. Click here for file

## References

[B1] Adler R (2000). A model of retinal cell differentiation in the chick. Prog Ret Eye Res.

[B2] Austin CP, Feldman DE, Ida JA, Cepko CL (1995). Vertebrate retinal ganglion cells are selected from competent progenitors by the action of Notch. Development.

[B3] Sadzinski LM, Matter JM, Ong MT, Hernandez J, Ballivet M (2001). Specification of neurotransmitter receptor identity in developing retina: the chick ATH5 promoter integrates the positive and negative effects of several bHLH proteins. Development.

[B4] Liu W, Khare SL, Liang X, Peters MA, Liu X, Cepko CL, Xiang M (2000). All Brn3 genes can promote retinal ganglion cell differentiation in the chick. Development.

[B5] Oblander SA, Ensslen-Craig SE, Longo FM, Brady-Kalnay SM (2007). E-cadherin promotes retinal ganglion cell neurite outgrowth in a protein tyrosine phosphatase-mu-dependent manner. Mol Cell Neurosci.

[B6] Walkenhorst J, Dutting D, Handwerker C, Huai J, Tanaka H, Drescher U (2000). The EphA4 receptor tyrosine kinase is necessary for the guidance of nasal retinal ganglion cell axons in vitro. Mol Cell Neurosci.

[B7] Philipsborn CV, Lang S, Loeschinger J, Bernard A, David C, Lehnert D, Bonhoeffer F, Bastmeyer M (2006). Growth cone navigation in substrate-bound ephrin gradients. Development.

[B8] Sakagami K, Ishii A, Shimada N, Yasuda K (2003). RaxL regulates chick ganglion cell development. Mech Dev.

[B9] Turner BA, Sparrow J, Cai B, Monroe J, Mikawa T, Hempstead BL (2006). TrkB/BDNF signaling regulates photoreceptor progenitor cell fate decisions. Dev Biol.

[B10] Moreira EF, Adler R (2006). Effects of follistatin overexpression on cell differentistion in the chick embryo retina. Dev Biol.

[B11] Godbout R, Marusyk H, Bisgrove D, Dabbagh L, Poppema S (1995). Localization of a fatty acid binding and its transcript in the developing chick retina. Exp Eye Res.

[B12] Bisgrove DA, Godbout R (1999). Differential expression of AP-2α and AP-2β in the developing chick retina: repression of R-FABP promoter activity by AP-2. Dev Dynamics.

[B13] Bisgrove DA, Monckton EA, Godbout R (1997). Involvement of AP-2 in regulation of the R-FABP gene in the developing chick retina. Mol Cell Biol.

[B14] Godbout R (1993). Identification and characterization of transcripts present at elevated levels in the undifferentiated chick retina. Exp Eye Res.

[B15] Fuhrman S, Levine EM, Reh TA (2000). Extraocular mesenchyme patterns the optic vesicle during early eye development in the embryonic chick. Development.

[B16] Rodriguez J, Esteve P, Weinl C, Ruiz JM, Fermin Y, Trousse F, Dwivedy A, Holt C, Bovolenta P (2005). SFRP1 regulates the growth of retinal ganglion cell axons through Fz2 receptor. Nat Neurosci.

[B17] Qian JQ, Jiang Z, Li M, Heaphy P, Liu YH, Shackleford GM (2003). Mouse *Wnt9b *transforming activity, tissue-specific expression, and evolution. GENOMICS.

[B18] Bergstein I, Eisenbreg LM, Bhalerao J, Jenkins NA, Copeland NG, Osborne MP, Bowcock AM, Brown AMC (1997). Isolation of two novel WNT Genes, WNT14 and WNT15, one of which (WNT15) is closely linked to WNT3 on human chromosome 17q21. GENOMICS.

[B19] Osakabe F, Ooto S, Akagi T, Mandai M, Akaike A, Takahashi M (2007). Wnt signaling promotes regeneration in the retina of adult mammals. J Neurosci.

[B20] Cho SH, Cepko CL (2006). Wnt2b/β-catenin-mediated canonical Wnt signaling determines the peripheral Fates of the chick eye. Development.

[B21] Nishida T, Kaneko F, Kitagawa M, Yasuda H (2001). Characterization of a novel mammalian SUMO-1/Smt3-specific is opeptidase, a homologue of rat AXAM, which is an axin-binding protein promoting β-catenin degradation. J Biol Chem.

[B22] Yamaguchi M, Tonou-Fujimori N, Komori A, Maeda R, Nojima Y, Li H, Okamoto H, Masai I (2005). Histone deacetylase 1 regulates retinal neurogenesis in zebrafish by suppressing Wnt and Notch signaling pathways. Development.

[B23] Romm E, Nielsen JA, Kim JG, Hudson LD (2005). Myt1 family recruits histone deacetylase to regulate neural transcription. J Neurochem.

[B24] Jehuda P, Sepkuty JP, Cohen AS, Eccles C, Rafiq A, Behar K, Ganel R, Coulter DA, Rothstein JD (2002). A neuronal glutamate transporter contributes to neurotransmitter GABA synthesis and epilepsy. J Neurosci.

[B25] Catsicas M, Mobbs P (2001). GABA-B receptors regulate chick retinal calcium waves. J Neurosci.

[B26] Catsicas M, Allcorn S, Mobbs P (2001). Early activation of Ca(2+)-permeable AMPA receptors reduces neurite outgrowth in embryonic chick retinal neurons. J Neurobiol.

[B27] Catsicas M, Bonness V, Becker D, Mobbs P (1998). Spontaneous Ca2+ transients and their transmission in the developing chick retina. Cur Biol.

[B28] Wong WT, Sanes JR, Wong ROL (1998). Developmentally regulated spontaneous activity in the embryonic chick retina. J Neurosci.

[B29] Hering H, Kroger S (1996). Formation of synaptic specializations in the inner plexiform layer of the developing chick retina. J Comp Neurol.

[B30] Hu M, Bruun A, Ehinger B (1998). The expression of GABA(A) receptors during the development of the rabbit retina. Acta Ophthalmol Scand.

[B31] Mitchell CK, Redburn DA (1996). GABA and GABA-A receptors are maximally expressed in association with cone synaptogenesis in neonatal rabbit retina. Dev Brain Res.

[B32] Karne A, Oakley DM, Wong GK, Wong RO (1997). Immunocytochemical localization of GABA, GABAA receptors, and synapse-associated proteins in the developing and adult ferret retina. Vis Neurosci.

[B33] Croci C, Brandstatter JH, Enz R (2003). ZIP3, a new splice variant of the PKC-ζ-interacting protein family, binds to GABAc receptors, PKC-ζ, and Kvβ2. J Biol Chem.

[B34] Kang X, Chong J, Ni M (2005). HYPERSENSITIVE TO RED AND BLUE 1, a ZZ-Type zinc finger protein, regulates phytochrome B-mediated red and cryptochrome-mediated blue light responses. Plant Cell.

[B35] Weishaupt JH, Diem R, Kermer P, Krajewski S, Reed JC, Bahr M (2003). Contribution of caspase-8 to apoptosis of axotomized rat retinal ganglion cells in vivo. Neurobiol Dis.

[B36] Neekhra A, Luthra S, Chwa M, Seigel G, Gramajo AL, Kuppermann BD, Kenney MC (2007). Caspase-8, -12, and -3 activation by 7-ketocholesterol in retinal neurosensory cells. Invest Ophthalmol Vis Sci.

[B37] Kim HS, Park CK (2005). Retinal ganglion cell death is delayed by activation of retinal intrinsic cell survival program. Brain Res.

[B38] Zwaan J, Wang L, Garza A, Lam KW (1994). Neuron-specific enolase expression during eye development in the chicken embryo. Exp Eye Res.

[B39] Lokhov PG, Moshkovskii SA, Ipatova OM, Prozorovskii VN (2004). Cytosolic insulin-binding proteins of mouse liver cells. Protein Pept Lett.

[B40] Kozielski F, Sack S, Marx A, Thormahlen M, Schonbrunn E, Biou V, Thompson A, Mandelkow EM, Mandelkow E (1997). The crystal structure of dimeric kinesin and implications for microtuble-dependent motility. Cell.

[B41] Bost-Usinger L, Chen RJ, Hillman D, Park H, Burnside B (1997). Multiple kinesin family members expressed in teleost retina and RPE include a novel C-terminal kinesin. Exp Eye Res.

[B42] Whitehead JL, Wang SY, Bost-Usinger L, Hoang E, Frazer KA, Burnside B (1999). Photoreceptor localization of the KIF3A and KIF3B subunits of the heterotrimeric microtubule motor kinesin ll in vertebrate retina. Exp Eye Res.

[B43] Whited JL, Cassell A, Brouillette M, Garrity PA (2004). Dynactin is required to maintain nuclear position within postmitotic Drosophila photoreceptor neurons. Development.

[B44] Pramatarova A, Ochalski PG, Lee CH, Howell BW (2006). Mouse Disabled 1 regulates the nuclear position of neurons in a *Drosophila *eye model. Mol Cell Biol.

[B45] Louis S, Ramagli, Link AJ (1999). Quantifying protein in 2-D page solubilization buffers. Methods in Molecular Biology.

[B46] Kanamoto T, Hellman U, Heldin CH, Souchelnytskyi S (2002). Functional proteomics of transforming growth factor-beta-1stimulated Mv1Lu epithelial cells: Rad51 as a target of TGF beta1-dependent regulation of DNA repair. EMBO J.

