# Supplementary figures and images for: Proteome profiling of embryo chick retina
Source: Proteome Sci. 2008 Jan 22;6:3. doi: 10.1186/1477-5956-6-3 (PMC2267454; doi:10.1186/1477-5956-6-3)

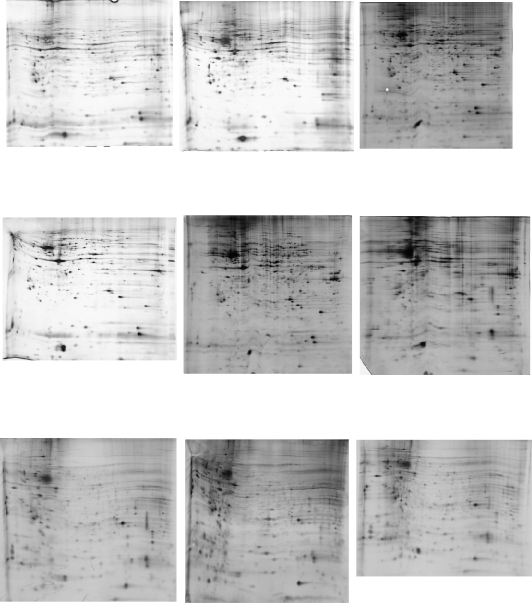

Supplement: Additional file 1 — Actual photographs of two-dimensional electrophoresis gels. Upper three panels showed silver-stained gels from ED7, middle from ED11, and lower from ED15. [file 1477-5956-6-3-S1.BMP]
