# Supplementary material for: Proteome profiling of embryo chick retina
Source: Proteome Sci. 2008 Jan 22;6:3. doi: 10.1186/1477-5956-6-3 (PMC2267454; doi:10.1186/1477-5956-6-3)
Supplement: Additional file 2 — Protein volume of significant spots. The data provide the actual protein volume (arbitary unit; a.u.) and SEMs of significant protein spots, measured by PD-Quest software. [file 1477-5956-6-3-S2.pdf]

	ED7	(SEM)	ED11	(SEM)	ED15	(SEM)
S1	0	0	20708	(6013)	3018	(5409)
S2	385	(102)	5346	(1325)	1788	(520)
S3	670	(207)	6464	(2000)	784	(2557)
S4	3145	(312)	7551	(2798)	7269	(450)
S5	11789	(2636)	26450	(4146)	19789	(2935)
S6	6518	(695)	3082	(446)	916	(1130)
S7	10576	(1801)	4297	(989)	7517	(1387)
S8	11596	(2641)	4737	(637)	7973	(1025)
S9	13066	(5311)	3139	(501)	5178	(3972)
S10	7198	(1502)	958	(98)	4012	(674)
S11	9433	(3126)	3340	(649)	13645	(1249)
S12	4152	(936)	11996	(3633)	0	0
S13	12134	(2690)	27681	(6235)	48942	(8493)
